# Synergistic improvement of high glyphosate resistance and high yield with environmental safety assessment

**DOI:** 10.3389/fpls.2026.1936824

**Published:** 2026-09-02

**Authors:** Peng Wang, Mengmeng Li, Juyun Zheng, Jiahui Liu, Jingjing Zhan, Yadi Xing, Yan Zhao, Wenshuai Zhang, Xiaoyang Ge, Xueyuan Li, Quanjia Chen, Fuguang Li

**Affiliations:** 1 Engineering Research Centre of Cotton, Ministry of Education/College of Agriculture, Xinjiang Agricultural University, Urumqi, China; 2 State Key Laboratory of Cotton Bio-breeding and Integrated Utilization, Institute of Western Agricultural of CAAS, Changji, Xinjiang, China; 3 Xinjiang Key Laboratory of Cotton Genetic Improvement and Intelligent Production/Cotton Research Institute of Xinjiang Academy of Agricultural Sciences, Urumqi, Xinjiang, China; 4 Anyang High and New Zone Entrepreneurship Service Center, Anyang, Henan, China; 5 Anyang Institution of Sino-cotton Industry Innovation, Anyang, Henan, China

**Keywords:** cotton, environmental safety assessment, fiber quality, glyphosate resistance, high yield

## Abstract

Glyphosate, one of the most widely used and effective herbicides worldwide, plays a critical role in weed management. However, its application can indirectly compromise crop yield and fiber quality while controlling weeds. Consequently, breeding crop varieties that simultaneously exhibit high herbicide tolerance, high yield, and excellent quality has become a major goal in current crop breeding. To address this critical gap, we successfully developed a transgenic upland cotton line, designated as L397-1, by co-transforming the elite cultivar ZM24 with two functional genes: the herbicide-resistant *g10evo-epsps* and the high-yield *csRRM2*. Phenotypic analysis showed that L397–1 plants exhibited significantly enhanced tolerance to glyphosate, as well as increased yield and fiber length, indicating that this strategy effectively pyramids high-yield, high-quality, and high-herbicide-resistance traits. Furthermore, comprehensive environmental safety assessments were conducted on the transgenic plants, covering their competitive fitness in both wasteland and cultivated habitats, field arthropod community structure, incidence of major diseases, and the population density and species richness of harmful pests. The results revealed no significant differences between the transgenic L397–1 and its recipient ZM24 across all evaluated ecological parameters, supporting the ecological safety and application potential of L397–1 for future breeding utilization. In summary, this study establishes an integrated chain from “trait pyramiding technology development—germplasm innovation—multidimensional evaluation of breeding value”, providing new insights for the synergistic improvement of multiple traits and their utilization in crop breeding.

## 1 Introduction

Weed infestation, one of the three major biological disasters in agricultural production, not only depletes essential soil nutrients such as nitrogen, phosphorus, potassium, and micronutrients but also blocks sunlight, thereby affecting photosynthesis, leaf surface temperature, and ultimately compromise both crop quality and yield (Heap and Duke, 2018; Bacmaga et al., 2024). According to statistics, weeds cause approximately 10–15% of annual global losses in major cereal crops, with an estimated global economic loss of about $40 billion per year. Currently, the most effective method for weed control is the application of herbicides at appropriate dosages. For instance, glyphosate—the most widely used herbicide—kills weeds by inhibiting the shikimate pathway and aromatic amino acid biosynthesis, thereby mitigating the impact of weeds on crop production (Heap and Duke, 2018). However, studies have shown that while herbicides control weeds, they can also cause adverse effects on crop quality and yield. Consequently, enhancing crop tolerance to herbicides has become a major bottleneck constraining the sustainable development of modern agriculture.

Studies have revealed that glyphosate primarily targets and inhibits 5-enolpyruvylshikimate-3-phosphate synthase (EPSPS), a key enzyme in the aromatic amino acid biosynthesis pathway (Sammons and Gaines, 2014; Chinnadurai et al., 2018). This inhibition disrupts the biosynthesis of aromatic amino acids, thereby impairing normal protein synthesis and leading to plant death (Hollander and Amrhein, 1980; Richards et al., 2006). Based on this mechanism, recent research has focused on two main molecular strategies to enhance herbicide tolerance in plants. The first strategy involves modifying the glyphosate-binding domain of EPSPS via protein engineering to reduce its affinity for the herbicide. The second strategy entails screening for heterologous EPSPS genes from naturally resistant organisms. When endogenous EPSPS is inactivated by glyphosate, the introduced functional exogenous EPSPS can sustain the biosynthesis of aromatic amino acids, ensuring plant survival and growth under herbicide treatment. This gene compensation mechanism effectively confers glyphosate tolerance in crops. Utilizing this strategy, numerous genes, including *aroA* (Comai et al., 1983), *CP4-epsps* (Chinnadurai et al., 2018; Funke et al., 2006; Kuiper et al., 2001), *G2-epsps* (Guo et al., 2015), and *GR79-epsps* (Liang et al., 2017), have been successively isolated and characterized for the development of transgenic glyphosate-resistant crops. Despite significant progress in enhancing glyphosate resistance in crops, breeding goals aimed at simultaneously improving both glyphosate tolerance and crop yield remains elusive.

In recent years, advances in research methodologies for the synergistic improvement of multiple traits have provided novel insights into the pyramiding of high-yield and high-resistance traits. For example, one strategy involves identifying pleiotropic factors that simultaneously regulate both high yield and stress tolerance, thereby achieving the aggregation of these two desirable traits (Fang et al., 2019; Qi et al., 2024; Wei et al., 2022; Zhao et al., 2023a). Although a small number of such pleiotropic factors that concurrently enhance yield and stress resistance have been identified, most of the discovered pleiotropic genes merely strengthen a single trait (either stress resistance or high yield) while inhibiting another desirable trait (De Lange et al., 2018). This trade-off significantly limits their practical value in breeding applications (Fan et al., 2014). Recently, a multi-gene co-transformation technology has been developed, which integrates multiple genes into single expression vector and successfully generates transgenic plants with the pyramiding of several desirable traits. Nevertheless, how to efficiently pyramid high glyphosate resistance and high yield traits in cotton remains unclear.

In this study, using our previously established multi-gene co-transformation platform, we successfully introduced both the herbicide-resistant *g10evo-epsps* and the high-yield *csRRM2* genes into the upland cotton variety Zhongmiansuo 24 (ZM24). The resulting transgenic cotton line, designated as L397-1, was subjected to phenotypic analysis, which revealed significant improvements in glyphosate tolerance, yield, and fiber length. To further evaluate its breeding value, we conducted a comprehensive environmental safety assessment of the transgenic L397-1, focusing on competitive ability (including in wasteland and cultivated fields) and biodiversity. The results showed no significant differences between L397–1 and ZM24 across these ecological parameters, indicating that the transgenic cotton line L397–1 exhibits comparable ecological characteristics to the recipient variety under tested conditions. In summary, this study not only provides a new insight for alleviating the trade-off between stress tolerance and yield but also offers valuable germplasm resources for developing new transgenic cotton cultivars with high yield and herbicide resistance.

## 2 Materials and methods

### 2.1 Plant materials and experimental design

Upland cotton (Gossypium hirsutum L.) cv. Zhongmiansuo 24 (ZM24, also designated CCRI24) was used as the transformation recipient. The transgenic lines and their genetic backgrounds are summarized in **Table 1**. Field trials were conducted at the experimental base of Anyang Institution of Sino-cotton Industry Innovation (Anyang, Henan, China) in 2023 and 2024. The experiment was arranged in a randomized complete block design with three independent biological replicates (plots). Each plot covered an area of 20 m² (4 m × 5 m), with a row spacing of 0.76 m and a plant spacing of 0.25 m. Greenhouse experiments were performed under controlled conditions (28 °C day/25 °C night, 16 h light/8 h dark photoperiod, 60% relative humidity) at the same institute in 2023.

**Table 1:** Description of cotton materials used in this study.

Line name	Genetic background	Transgenes	Promoter	Generation	Event type
ZM24 (CCRI24)	Gossypium hirsutum cv. Zhongmiansuo 24	None	–	–	Non-transgenic recipient control
A-L397-1	ZM24	g10evo-epsps	CaMV 35S	T4	Single-gene transgenic event
B-L397-1	ZM24	csRRM2	CaMV 35S	T4	Single-gene transgenic event
L397-1	ZM24	g10evo-epsps + csRRM2	CaMV 35S (each gene cassette)	T4	Double-gene pyramiding transgenic event

All transgenic field trials were approved by the Ministry of Agriculture and Rural Affairs of the People’s Republic of China. Field trials were conducted with standard isolation distances, and all residual plant materials were disposed of in accordance with national biosafety regulations.

### 2.2 Glyphosate tolerance assay

A gradient glyphosate treatment assay was conducted at the 4–6 true leaves seedling stage of cotton (25–30 days after sowing). The commercial glyphosate formulation used was 41% glyphosate isopropylamine salt, and all doses are expressed as grams of active ingredient per hectare (g a.i./ha). Glyphosate was uniformly applied using a backpack sprayer (operating pressure: 3 kg/cm²; spray volume: 30 L/ha) at various concentrations: 0× (0 g a.i./ha), 1× (82 g a.i./ha), 2× (164 g a.i./ha), and 4× (328 g a.i./ha) for the primary tolerance assay. For the high-dose tolerance assay, an extended gradient of 6× (492 g a.i./ha), 8× (656 g a.i./ha), 10× (820 g a.i./ha), 12× (984 g a.i./ha), 14× (1148 g a.i./ha), 16× (1312 g a.i./ha), and 18× (1476 g a.i./ha) was applied.

Phenotypic observations were recorded at three key time points post-application: 1, 2, and 4 weeks. Plant survival rates were evaluated by counting 25 tagged plants per plot, with three independent plots per treatment. Phytotoxicity symptoms were assessed at 2 and 4 week post-application using a 0–5 visual rating scale, where 0 = no symptoms, 1 = slight yellowing at leaf margins, 2 = yellowing of 50% of the leaves, 3 = malformation of new leaves, 4 = necrosis of the apical meristem, and 5 = plant death.

### 2.3 Vector construction and genetic transformation

A specific fragment covering the RRM2 domain within the coding region of the Brassica napus FCA gene (Hong et al., 2007) was amplified by PCR. The amplified fragment was then inserted into the expression vector pBin438 via double digestion with BglII and BstEII. Using the recombinant vector pBin438-csRRM2 as a template, the expression cassette was amplified by PCR. One redundant EPSPS expression construct in the modified pCAMBIA1300 vector was excised with HindIII and EcoRI. This expression cassette was then ligated into the HindIII/EcoRI-modified pCAMBIA1300/g10evo-EPSPS vector to generate pCAMBIA1300/g10evo-EPSPS-csRRM2. Separately, the same expression cassette was ligated into the unmodified pCAMBIA1300 vector to generate pCAMBIA1300/csRRM2. All primers used for PCR amplification were synthesized by Sangon Biotech (Shanghai) Co., Ltd., and their sequences are listed in **Supplementary Table 1**.

The binary vector was introduced into Agrobacterium tumefaciens strain LBA4404 via the freeze-thaw method. Genetic transformation was performed using hypocotyl explants of ZM24. After co-cultivation, explants were selected on MS medium supplemented with 50 mg/L kanamycin as the selectable marker. Resistant calli were induced to differentiate into embryogenic calli and regenerated into whole plantlets. Putative transgenic plants were screened by PCR amplification of the target genes. Homozygous lines were identified by segregation ratio analysis of the T2 and T3 generations via PCR and glyphosate resistance screening, and the T4 generation homozygous line L397–1 was used for subsequent experiments. The transformation efficiency of this method is approximately 2.3%.

### 2.4 Whole-genome resequencing

Whole-genome resequencing of the transgenic cotton line L397–1 was conducted on the BGISEQ500 platform. The average sequencing depth of the cotton genome was approximately 30×, with a genome mapping rate exceeding 98%. The workflow comprised the following steps: extraction of sequencing reads harboring the vector sequence; verification of T-DNA region integrity via sequence alignment; and alignment of vector-genome chimeric reads against the *Gossypium hirsutum* reference genome (NAUNBI version) to precisely determine the insertion site of the exogenous DNA in the genome and characterize its flanking sequences. The detailed procedures are as described in previous reports (Yang et al., 2026).

### 2.5 Southern blotting

Southern blot analysis was performed using digoxigenin (DIG)-labeled probes. Genomic DNA was extracted from young leaves of transgenic cotton plants. For each sample, 20 μg of genomic DNA was digested completely with *Eco*RI or *Bam*HI (neither enzyme has a recognition site within the T-DNA region) overnight at 37 °C, separated by agarose gel electrophoresis, and transferred onto a positively charged nylon membrane via capillary blotting, followed by UV cross-linking for DNA immobilization.

**Figure 1:**
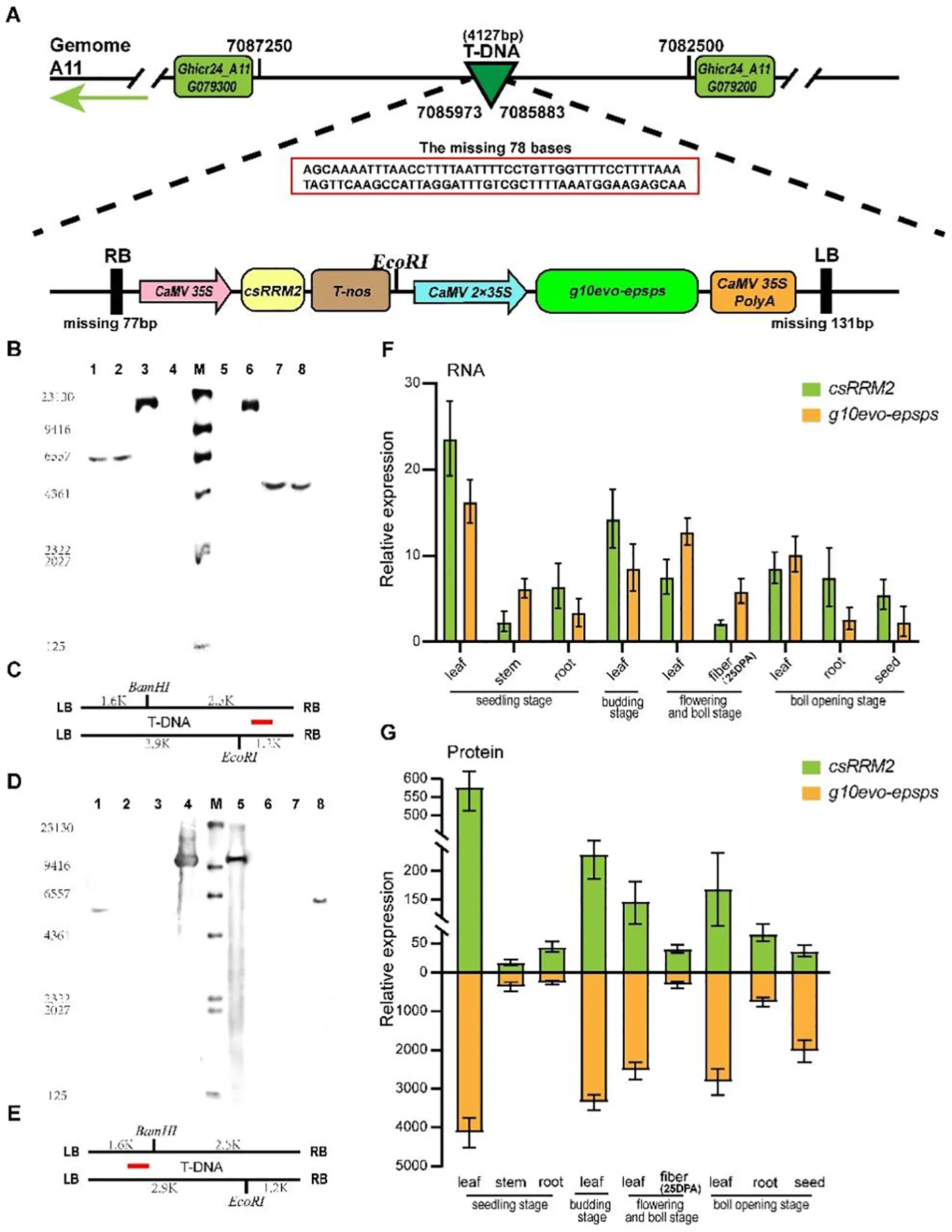
Molecular characterization of transgenic cotton L397-1. **(A)** Schematic diagram illustrating the T-DNA insertion site in the genome of transgenic cotton L397-1. **(B)** Southern blot analysis of the *csRRM2* gene. Genomic DNA was digested with *Eco*RI and *Bam*HI. Lane M: DNA marker; Lanes 1–4: *Bam*HI-digested DNA from T3 generation L397-1, T4 generation L397-1, plasmid, and CCRI24, respectively; Lanes 5–8: *Eco*RI-digested DNA from CCRI24, plasmid, T3 generation L397-1, and T4 generation L397-1, respectively. **(C)** Schematic representation of the *csRRM2* probe position and restriction enzyme cleavage sites. The horizontal line indicates the probe position, and the vertical lines indicate the restriction sites. **(D)** Southern blot analysis of the *g10evo-epsps* gene following digestion with *Eco*RI and *Bam*HI. Lane M: DNA marker; Lanes 1–4: *Bam*HI-digested DNA from T4 generation L397-1, blank control, CCRI24, and plasmid, respectively; Lanes 5–8: *Eco*RI-digested DNA from plasmid, CCRI24, blank control, and T4 generation L397-1, respectively. **(E)** Schematic representation of the *g10evo-epsps* probe position and restriction enzyme cleavage sites. The horizontal line indicates the probe position, and the vertical lines indicate the restriction sites. **(F)** Transcriptional expression analysis of the target genes in L397-1. Data are presented as mean ± SD (n=3) **(G)** Translational expression analysis of the target genes in L397-1. Data are presented as mean ± SD (n=3).

Probes were prepared by PCR amplification of target sequences and labeled with DIG using the Roche DIG High Prime DNA Labeling Kit (Cat. No. 11745832910) according to the manufacturer’s instructions. The probe positions and expected fragment sizes are shown in **Figures 1C, E**. The membrane was prehybridized and then hybridized with the DIG-labeled probe in hybridization buffer. After hybridization, the membrane was sequentially washed, blocked, and incubated with anti-DIG alkaline phosphatase conjugate. Detailed procedures were carried out as described in the kit manual. A single hybridizing band of the expected size was observed for each probe, supporting single-copy integration of the T-DNA.

### 2.6 RNA isolation and quantitative real-time PCR analysis

Total RNA was isolated from cotton tissues using the RNAprep Pure Plant Kit. RNA quality was assessed by agarose gel electrophoresis and A260/A280 ratio (1.9–2.1). First-strand cDNA was synthesized using the PrimeScript™ RT Reagent Kit with gDNA Eraser. RT-qPCR was performed on an ABI 7900 Real-Time PCR System using SYBR^®^ Premix Ex Taq™.

The cotton *Histone3* gene was used as the internal reference gene. Primer efficiencies were validated via standard curve analysis, with efficiencies ranging from 95% to 105%. Melting curve analysis was performed to confirm amplification specificity. No-template and no-reverse-transcriptase controls were included in each run. The experiments included three biological replicates, each with three technical replicates. Relative expression levels were calculated using the 2^−ΔΔCT^ method. Primers used for the RT-qPCR assay are listed in **Supplementary Table 1**.

### 2.7 ELISA for protein quantification

Total soluble protein was extracted from frozen tissue samples using PBS buffer (pH 7.4) containing protease inhibitor cocktail. Protein concentration of each extract was determined by the Bradford method for sample normalization. All ELISA assays included three biological replicates per tissue type.

csRRM2 protein quantification was determined using an indirect ELISA. Microplates were coated with 100 μL/well of serially diluted recombinant csRRM2 standards (0.156–10 ng/mL) or appropriately diluted samples (1:10) and incubated overnight at 4 °C. Following three washes with PBST, wells were blocked with 200 μL/well of 5% skim milk-PBS for 1 h at 37 °C. After incubation with rabbit anti-csRRM2 polyclonal primary antibody (1:1000, 100 μL/well, 2 h at 37 °C) and HRP-conjugated goat anti-rabbit secondary antibody (1:5000, 100 μL/well, 1 h at 37 °C), the reaction was developed using TMB substrate (100 μL/well, 15 min in the dark at room temperature) and terminated with 50 μL of 2 M H_2_SO_4_. Absorbance was measured at 450 nm (reference: 630 nm). Protein concentrations were calculated via a four-parameter logistic regression model (R² ≥ 0.990). The polyclonal antibody was validated for specificity via Western blot analysis.

G10evo-EPSPS protein quantification was carried out using a highly specific double-antibody sandwich ELISA. High-binding plates were coated with anti-G10evo-EPSPS monoclonal antibody (2 μg/mL in PBS, pH 7.4, 100 μL/well) overnight at 4 °C. After washing with PBST, wells were blocked with 1% BSA-PBS (200 μL/well) for 2 h at 37 °C. Standard (0.1–10 ng/mL purified G10evo-EPSPS) and 1:5 diluted samples (100 μL/well) were added and incubated for 1.5 h at 37 °C. After five washes, HRP-conjugated detection antibody (1:3000, 100 μL/well) was added and incubated for 1 h at 37 °C. The signal was developed with ultrasensitive TMB substrate (100 μL/well) for 10 min in the dark, stopped with 2 M H_2_SO_4_ (50 μL/well), and read at 450/630 nm. A four-parameter logistic standard curve (R² ≥ 0.995, sensitivity 0.05 ng/mL) was used for quantification.

### 2.8 Pollen viability assessment

Transgenic line L397–1 and the control ZM24 were foliar-sprayed with water or glyphosate at 1×, 2×, 4×, 6×, and 8× field rates during the flowering period in July. Flowers were sampled at 1, 7, 14, and 21 days post-application (covering 3 consecutive weeks after treatment) between 9:00 and 10:00 a.m., with at least three biological replicates per treatment. Samples were immediately stored on ice.

Pollen viability was assessed using Alexander’s stain, which provides an indirect indicator of viability based on cytoplasmic integrity. Mature pollen was collected and washed 2–3 times with phosphate-buffered saline (PBS) to remove debris. The pollen was then stained with approximately 20 μL of Alexander’s stain at 25 ± 2 °C for 15–20 min. After staining, a coverslip was gently applied, and excess stain was blotted with filter paper. Viable pollen stained uniformly purplish-red and appeared plump, spherical or ellipsoidal. Aborted pollen was shrunken (length/width ratio >1.5) and showed abnormal staining (absent or partial).

For each sample, at least five fields of view (≥50 pollen grains per field) were examined under brightfield (100× for localization, 400× for counting). Images were captured with a digital microscope system (CCD camera), and the stained area ratio (purplish-red pixels/total pollen pixels) was analyzed using ImageJ. The percentage of viable pollen per field was calculated.

### 2.9 Fiber quality assessment

To systematically evaluate the fiber quality traits of different cotton materials, 10 g fiber samples were randomly collected from each treatment group in the experimental plots. All measurements were conducted by the Cotton Quality Supervision and Testing Center of the Ministry of Agriculture and Rural Affairs (CMA/CNAS accredited) using a standardized protocol. The quality parameters were determined using an HVI 1000 high-volume fiber testing instrument. This approach ensured high data accuracy and comparability.

### 2.10 Photosynthetic parameter measurement

Relative chlorophyll content (SPAD value) was measured on the third fully expanded leaf from the top using a SPAD-502 chlorophyll meter (Konica Minolta, Japan) at 1, 2, and 4 days after glyphosate treatment. The maximum photochemical efficiency of photosystem II (Fv/Fm) was determined using a Pocket PEA chlorophyll fluorometer (Hansatech, UK) after 30 min of dark adaptation. For each treatment, 20 individual plants were measured as biological replicates.

### 2.11 Competitive fitness assessment

The wasteland competition experiment was conducted on uncultivated abandoned land with natural weed vegetation. Two sowing methods (broadcast sowing and 3 cm deep sowing) were applied, with three replicate plots per treatment. Weed coverage and cotton coverage were surveyed at 5 time points after sowing using the diagonal five-point sampling method (1 m × 1 m quadrat per point).

The cultivated land competition experiment was conducted in normal farmland under conventional cultivation. Germination rate, plant height, and seed number per plant were investigated at corresponding growth stages, with 30 plants per plot and three replicate plots.

### 2.12 Arthropod community and disease survey

Arthropod surveys were conducted across 13 developmental stages from sowing to harvest, using the five-point sampling method in each plot. At each sampling point, 5 consecutive cotton plants were investigated, and all arthropods on the plants were identified and counted. Taxonomic identification was performed to the species or morphospecies level. Three biodiversity indices were calculated: (1) Shannon-Wiener diversity index: H′=−∑Pi​lnPi​, where Pi​ is the proportion of individuals of species I; (2) Simpson dominance index: D = 1−∑Pi2​; (3) Pielou evenness index: J=H′/lnS, where S is the total number of species.

Disease surveys were conducted at the seedling, squaring, flowering/bolling, and boll-opening stages. The incidence of boll rot was calculated as the percentage of diseased bolls. The disease index of Verticillium wilt was scored on a 0–4 scale based on leaf symptom severity and calculated as: Disease index = [Σ (disease grade × number of plants in grade)/(total plants × highest grade)] × 100. Each survey included 30 plants per plot with three replicate plots.

### 2.13 Statistical analyses

Statistical analyses were performed using GraphPad Prism 9.0 software. For pairwise comparisons between the transgenic line and the recipient control under the same treatment condition, a two-tailed Student’s t-test was used. For multi-dose and multi-timepoint experiments, pairwise comparisons were performed with Bonferroni correction for multiple testing where appropriate.

Data are presented as mean ± standard deviation (SD), with the number of biological replicates (n) specified in each figure legend. Significance levels were indicated as follows: *P < 0.05; **P < 0.01; ***P < 0.001; ****P < 0.0001. For comparisons among more than two groups, one-way ANOVA followed by Duncan’s multiple range test (*P* < 0.05) was used, and statistically significant differences are denoted by different letters.

## 3 Results

### 3.1 Generation of transgenic cotton L397–1 via multi-gene pyramiding and its molecular characterization

To achieve the efficient pyramiding of herbicide resistance and high-yield traits in cotton, we first constructed an overexpression vector, 35S:*g10evo-epsps*/*csRRM2*, using a multi-gene pyramiding technique. This vector was then genetically transformed into ZM24 cotton, with 35S:*csRRM2* and 35S:*g10evo-epsps* transformed lines serving as controls (**Supplementary Figure 1A**). Subsequently, seedling leaves were collected to extract DNA for PCR-based screening of positive transformants (**Supplementary Figure 1B**), and the positive plants were transplanted to the greenhouse. Homozygous positive lines were then subjected to molecular characterization and phenotypic evaluation.

Among the numerous 35S::*g10evo-epsps*/*csRRM2* positive lines, we identified a transgenic line exhibiting high glyphosate resistance, designated as L397-1. To further verify the integration of the T-DNA into the cotton genome, primers targeting the key functional elements (left and right flanking sequences, promoter, target gene, and terminator) were designed for PCR amplification and sequencing of the L397–1 plants (**Figure 1A**).

Sequence analysis revealed that the entire 4,127-bp T-DNA was inserted at positions 7,085,883–7,085,973 on chromosome A11 of the cotton genome in a forward orientation, with a 78-bp genomic deletion at the insertion site. The junction sequences at both the right border (RB) and left border (LB) were confirmed by Sanger sequencing. This locus resides in an intergenic region, with the insertion site located more than 1.0 kb away from both the upstream and downstream genes. A total of 32 junction-spanning reads supported the RB integration site and 28 reads supported the LB site, with an average sequencing depth of 28× at the insertion locus.

Subsequently, whole-genome sequencing (WGS) was performed to further validate these findings (**Supplementary Figure 2**), yielding a total of 56,241,783 raw reads and 16,872,534,900 raw bases. The obtained sequencing reads were mapped to both the T-DNA region and the vector backbone of the expression vector pCAMBIA1300-35S::*g10evo-epsps*/*csRRM2*. The results showed that no reads matched the vector backbone at the given sequencing depth, indicating that no backbone sequence was integrated into the recipient genome. Conversely, the sequencing reads perfectly matched the 4,127 bp T-DNA sequence, confirming the precise integration of the T-DNA into the recipient genome.

We further systematically characterized the insertion features of the *csRRM2* and *g10evo-epsps* genes in the transgenic cotton line L397–1 using Southern blot, RT-qPCR, and ELISA assays. The results indicated that the exogenous sequences were integrated as a single copy into the cotton genome (**Figures 1B–E**). RT-qPCR analysis revealed that both *csRRM2* and *g10evo-epsps* were expressed across various developmental stages and tissues in the L397-1, with the highest expression levels observed in seedling leaves and the lowest in cotton fibers (**Figure 1F**). Meanwhile, ELISA was employed to quantify the protein expression levels of the target genes across different stages and tissues (**Figure 1G**). The results consistently showed that both csRRM2 and G10evo-EPSPS proteins exhibited the highest expression in seedling leaves and the lowest in cotton fibers.

### 3.2 Transgenic L397–1 plants exhibit enhanced glyphosate tolerance in cotton

**Figure 2:**
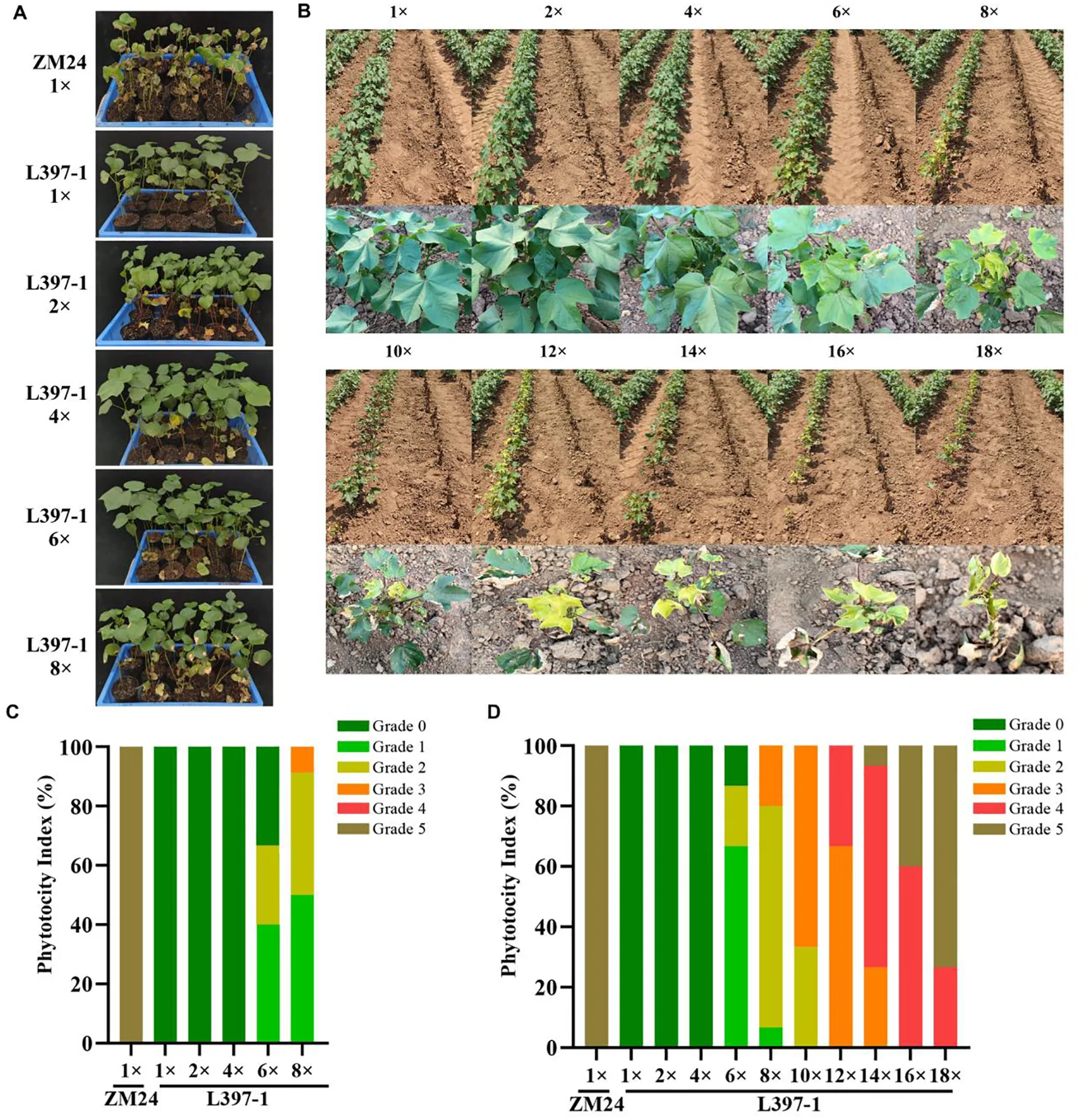
Enhanced glyphosate tolerance in cotton plants of line L397-1. **(A, B)** Field-grown seedling phenotypes **(A)** and survival statistics analysis **(B)** of L397–1 plants one week after treatment with varying concentrations of glyphosate. Each panel shows field phenotypes under different concentrations: transgenic line L397–1 on the left, Zhongmiansuo 24 (control) on the right (all died). The bottom insets present magnified views of the transgenic leaves at different concentrations. **(C, D)** Greenhouse-grown seedling phenotypes **(C)** and survival statistics **(D)** of L397–1 one week after treatment with varying concentrations of glyphosate. Phytotoxicity symptoms were assessed using grade 0–5 visual rating scale.

To evaluate the glyphosate tolerance of the L397–1 plants, we first sprayed the recipient material (ZM24) and the L397–1 line with various concentrations of glyphosate under greenhouse conditions. We found that the transgenic cotton L397–1 exhibited significantly higher glyphosate tolerance compared to the ZM24. Specifically, following the application of 1×, 2×, and 4× recommended field rates of glyphosate, the L397–1 plants achieved a 100% survival rate across all treatment groups, with no visible symptoms of phytotoxicity. In contrast, the ZM24 showed complete mortality after spraying with the 1× concentration, resulting in a survival rate of 0% (**Figures 2A, B**, **Table 1**).

Given that long-term use of herbicides often leads to weed resistance, higher doses are often applied in practice to achieve optimal weed control. To determine the upper limit of glyphosate tolerance in the L397–1 transgenic line, we applied ten concentration gradients of glyphosate (ranging from 1× to 18× the recommended field rate) to L397–1 plants grown under field conditions. Phytotoxicity phenotypes were evaluated one week post-treatment during the seedling stage (**Figures 2C, D**).

Phenotypic analysis revealed that all L397–1 transgenic plants survived across all ten concentration gradients, whereas the recipient plants (ZM24) died completely. Quantitative phytotoxicity grading based on the 0–5 scale showed that L397–1 plants maintained grade 0 (no symptoms) under 1× to 4× field rates, with no significant differences in seedling survival rate or plant height compared with untreated controls. At 6× and 8× field rates, the phytotoxicity grade increased to grade 1 and grade 2, respectively, manifested as slight leaf margin yellowing and partial leaf yellowing without growth arrest. When the concentration reached 10× to 12×, the phytotoxicity grade ranged from grade 2 to grade 3, accompanied by new leaf malformation. At the 14× concentration, the phytotoxicity grade reached grade 4, with apical meristem necrosis observed; however, the plants gradually recovered after one week, with new leaves emerging from lateral branches. Even at the highest concentration of 18×, all plants remained alive (grade 4, no plant death). These quantitative grading results consistently demonstrate that the transgenic plants possess exceptionally high resistance to glyphosate.

### 3.3 Photosynthetic performance and pollen viability under glyphosate treatment

**Figure 3:**
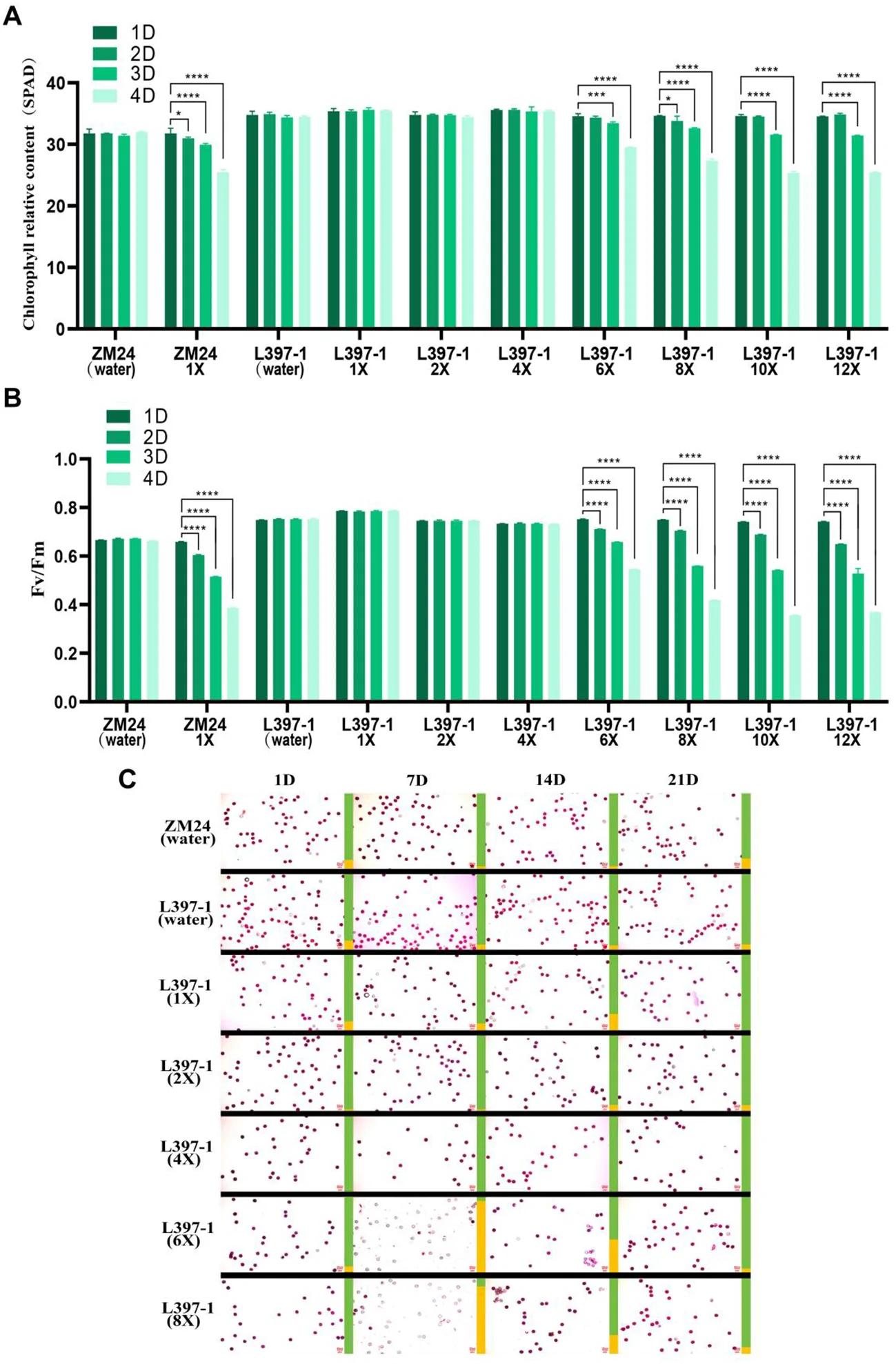
Physiological indices of transgenic cotton plants under glyphosate treatment. **(A)** Relative chlorophyll content of L397–1 and ZM24 after treatment with different concentrations of glyphosate (from day 1 to day 4). Values are shown as mean ± SD (*n* = 20). *P < 0.05; ***P < 0.001; ****P < 0.0001. **(B)** Maximum photosynthetic potential of L397–1 and ZM24 after treatment with different concentrations of glyphosate (from day 1 to day 4). Values are shown as mean ± SD (*n* = 20). ****P < 0.0001. **(C)** Pollen staining results of L397–1 and ZM24 over three consecutive weeks after treatment with different concentrations of glyphosate (high concentration of glyphosate affects the fertility of L397–1 plants).

To investigate the physiological response to glyphosate in L397-1, we first analyzed the photosynthetic characteristics of L397–1 and ZM24 under different glyphosate treatments (**Figures 3A, B**). Phenotypic analysis showed that there were no significant differences in relative chlorophyll content or maximum photochemical efficiency (Fv/Fm) between ZM24 and L397–1 under the water control treatment. Importantly, following treatment with the 1× glyphosate concentration, ZM24 exhibited a significant decrease in both relative chlorophyll content and Fv/Fm compared to the water control. In contrast, even under the 4× glyphosate treatment, the transgenic L397–1 plants showed no significant changes in these parameters relative to the water control. These results suggest that maintenance of photosynthetic performance is associated with the enhanced glyphosate tolerance observed in L397–1 plants.

Previous studies have shown that glyphosate application impairs pollen development and reduces viability by inhibiting multiple energy and metabolic pathways. To investigate whether the transgenic line L397–1 can restore pollen viability suppressed by glyphosate, we sprayed water or different concentrations of glyphosate on L397–1 and ZM24 plants, and collected pollen at 1, 7, 14, and 21 days after treatment for staining with Alexander’s stain (**Figure 3C**). Microscopic examination revealed that pollen viability in L397–1 plants was temporarily reduced one week after treatment with 6× field concentration of glyphosate, but viability gradually increased two weeks later and largely recovered by three weeks post-treatment. These results suggest that during field application of glyphosate, repeated high-concentration spraying during the flowering stage should be avoided to prevent adverse effects on fertility and consequent yield loss.

### 3.4 Transgenic line L397–1 enhances cotton yield and fiber quality

**Figure 4:**
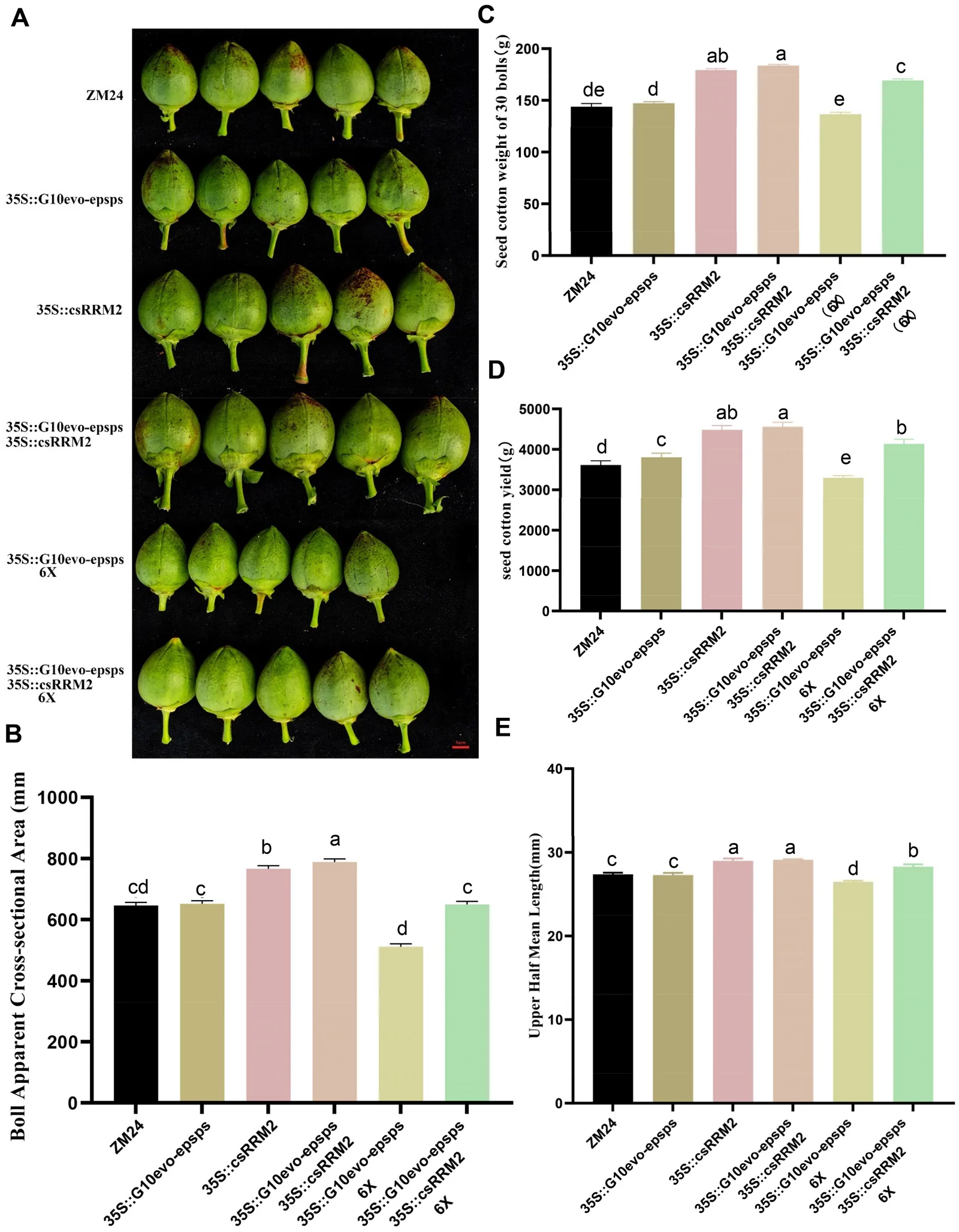
Transgenic line L397–1 enhances cotton yield and fiber quality. **(A, B)** Boll phenotypes **(A)** and boll size statistics **(B)** of various transgenic materials after glyphosate treatment. Values are shown as mean ± SD (*n* = 20). Different letters indicate statistically significant differences (one-way ANOVA with Duncan’s multiple range test, *P* < 0.05). **(C)** Seed cotton weight of various transgenic materials after glyphosate treatment. Different letters indicate statistically significant differences (one-way ANOVA with Duncan’s multiple range test, *P* < 0.05). **(D)** Seed cotton yield of various transgenic materials after glyphosate treatment. Different letters indicate statistically significant differences (one-way ANOVA with Duncan’s multiple range test, *P* < 0.05). **(E)** Fiber length of various transgenic materials after glyphosate treatment. Different letters indicate statistically significant differences (one-way ANOVA with Duncan’s multiple range test, *P* < 0.05).

To further evaluate the agronomic traits of the transgenic L397–1 plants beyond glyphosate resistance, we first measured the boll sizes of ZM24 and the L397–1 transgenic plants following glyphosate treatment (**Figures 4A, B**). For consistency, bolls were sampled from the first fruiting branch adjacent to the main stem on the same day. We found that the bolls of the 35S::*csRRM2* and L397–1 transgenic plants were significantly larger than those of ZM24 and the 35S::*g10evo-epsps* line. Notably, even after treatment with 6× the field concentration of glyphosate, L397–1 plants still produced larger bolls than untreated ZM24, highlighting the strong breeding potential of this transgenic line.

We further evaluated the seed cotton and lint cotton yields from 30 bolls per line (**Figures 4C, D**). The results demonstrated that both the seed cotton and lint yields of the 35S::*csRRM2* and L397–1 transgenic plants were significantly higher than those of ZM24 and 35S::*g10evo-epsps*. Collectively, these findings indicate that the overexpression of *csRRM2* significantly enhances both boll size and boll-level yield traits in the transgenic L397–1 plants.

To investigate whether the transgenic L397–1 plants affect cotton fiber quality, we measured the fiber length of four genotypes: ZM24, A-L397-1 (35S::*g10evo-epsps*), B-L397-1 (35S::*csRRM2*), and L397-1, under both untreated and glyphosate-treated conditions. Phenotypic analysis revealed that without glyphosate treatment, the fiber lengths of the ZM24 and A-L397–1 were significantly shorter than those of B-L397–1 and L397-1(**Figure 4E**). However, following treatment with 6× field concentration of glyphosate, the fiber length of L397–1 decreased to some extent but remained significantly higher than that of the controls. This indicates that L397–1 can improve cotton fiber length. Collectively, these findings demonstrate that the transgenic L397–1 plants effectively mitigate the trade-off between herbicide resistance and yield, achieving the efficient pyramiding of high resistance, high yield, and superior fiber quality. Thus, L397–1 holds tremendous potential for breeding applications.

### 3.5 Environmental safety assessment of the transgenic L397–1 line

**Figure 5:**
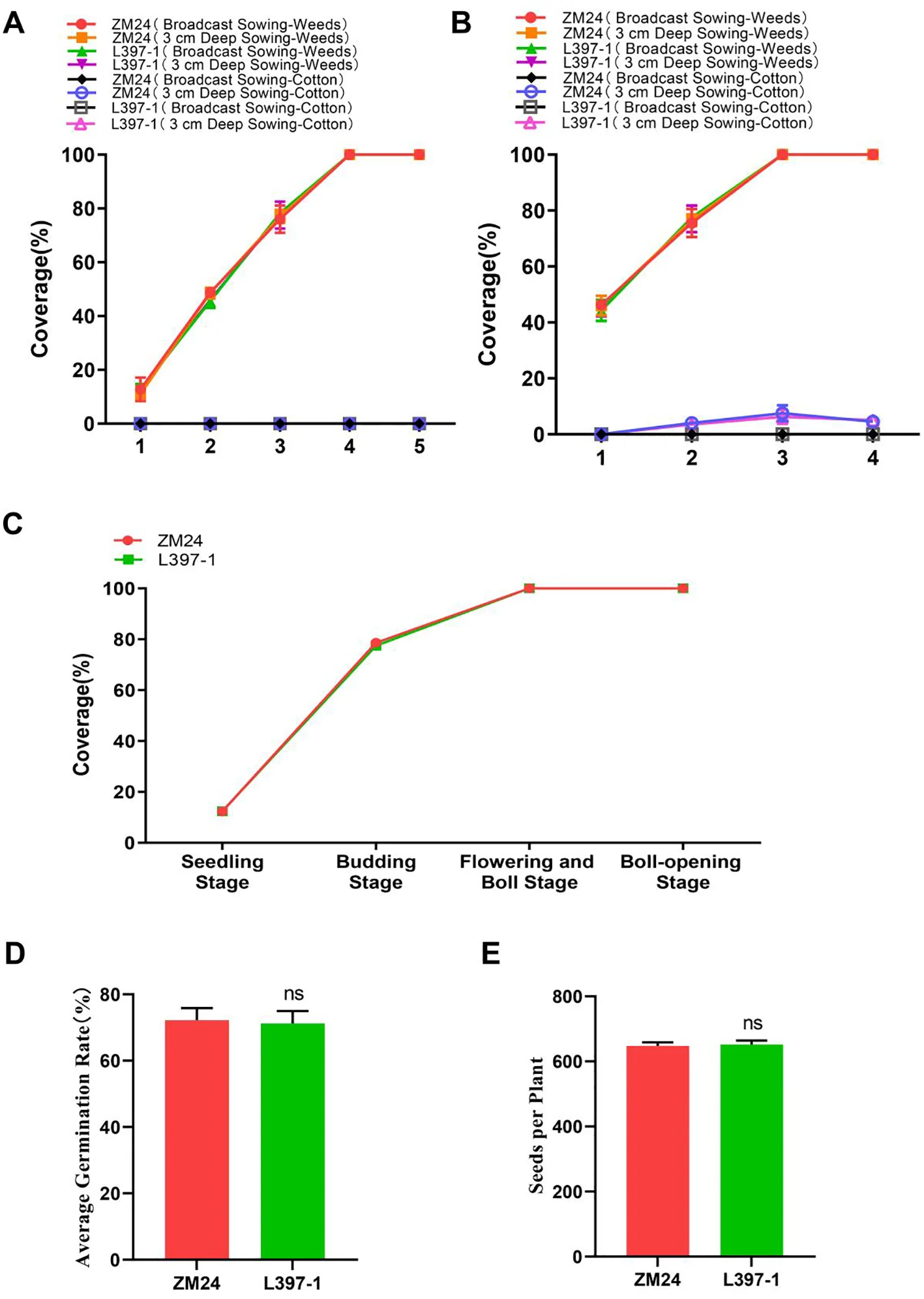
Assessment of the effect of L397–1 on survival competitiveness. **(A)** Coverage rate of L397–1 and ZM24 (control) after the first sowing period in uncultivated land. Values are shown as mean ± SD (*n* = 3). **(B)** Coverage rate of L397–1 and ZM24 (control) after the second sowing period in uncultivated land. Values are shown as mean ± SD (*n* = 3). **(C)** Coverage rate of L397–1 and ZM24 (control) after sowing in cultivated land. Values are shown as mean ± SD (*n* = 3). **(D)** Average germination rate of L397–1 and ZM24 (control) in cultivated land. **(E)** Number of seeds per plant of L397–1 and ZM24 (control) in cultivated land.

Assessing the survival and competitive ability of transgenic materials in uncultivated land is crucial for predicting potential weediness risks. Therefore, we first evaluated the survival competitiveness of the transgenic line L397–1 under uncultivated field conditions. We found that under the same sowing date and sowing method, the weed species composition was generally consistent across all treatment plots. The weed coverage in plots planted with transgenic cotton L397–1 showed no significant difference from that in plots planted with ZM24 control across all survey periods. Similarly, the cotton coverage of L397–1 was not significantly different from that of the ZM24 control under the same sowing date and sowing method across all survey periods (**Figures 5A–C**).

Subsequently, we analyzed the coverage of L397–1 at different growth stages and found no significant difference compared with the ZM24 control. Furthermore, statistical analysis of the average germination rate and seed number per plant in cultivated land revealed no significant differences between L397–1 and ZM24 control (**Figures 5D, E**). Collectively, these results indicate that the transgenic line L397–1 does not exhibit enhanced survival or competitive ability in uncultivated or cultivated habitats.

**Figure 6:**
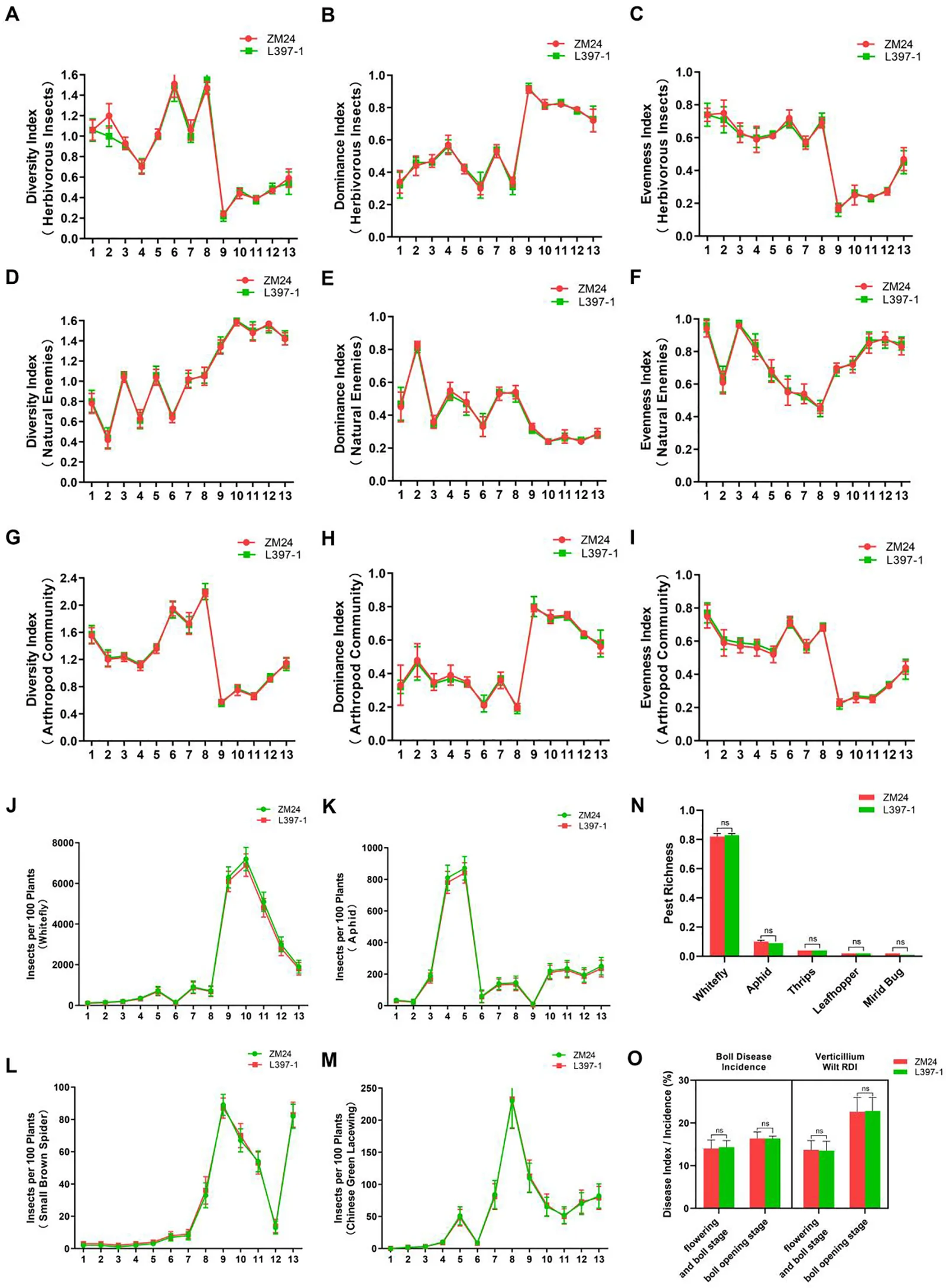
Assessment of the effect of L397–1 on biodiversity in cotton fields. **(A–C)** Biodiversity index **(A)**, dominance concentration index **(B)**, and evenness index **(C)** of herbivorous insects surveyed across 13 stages from sowing to harvest. **(D–F)** Biodiversity index **(D)**, dominance concentration index **(E)**, and evenness index **(F)** of natural enemies surveyed across 13 stages from sowing to harvest. **(G–I)** Biodiversity index **(G)**, dominance concentration index **(H)**, and evenness index **(I)** of arthropods surveyed across 13 stages from sowing to harvest. **(J–M)** Population dynamics of major herbivorous insects in cotton fields across 13 stages from sowing to harvest, including whitefly **(J)**, aphid **(K)**, *Tetranychus cinnabarinus* **(L)**, and *Chrysoperla sinica* **(M)**. **(N)** Analysis of the impact of transgenic cotton L397–1 on the pest population density and species richness. **(O)** Statistical analysis of disease incidence in transgenic cotton L397-1.

Subsequently, we investigated the effects of the L397–1 transgenic plants on the arthropod community structure in cotton fields across 13 developmental stages from sowing to harvest. We found that under the non-herbicide treatment, there were no significant differences in the biodiversity indices of phytophagous insects, natural enemies, and the total arthropod community between the transgenic L397–1 and the ZM24 plants (**Figures 6A–I**). Concurrently, we systematically monitored the population dynamics of various taxa to further evaluate the impact of the L397–1 line on the field arthropod community structure. No significant differences were observed in the population densities of all monitored groups—including whitefly, aphid, small brown spider, and green lacewing—between L397–1 and ZM24 (**Figures 6J–M**).

An analysis of the pest species richness of major harmful organisms in the cotton fields of L397–1 and ZM24 revealed no significant differences between the L397–1 transgenic line and the ZM24 control. Therefore, the transgenic L397–1 cotton did not significantly affect the pest population density and species richness in the cotton fields (**Figure 6N**).

Furthermore, we investigated the occurrence of diseases in both the transgenic L397–1 and ZM24 cotton plants during the seedling, squaring, flowering/bolling, and boll-opening stages. No diseases were detected during the seedling and squaring stages. During the flowering/bolling and boll-opening stages, the primary diseases observed were boll rot and Verticillium wilt. Further analysis of these two diseases showed no significant differences in the incidence of boll rot or the disease index of Verticillium wilt between the transgenic L397–1 line and ZM24 (**Figure 6O**). Taken together, these multi-dimensional evaluation results indicate that transgenic line L397–1 does not pose detectable additional ecological risks compared with the recipient variety ZM24 under the tested field conditions, providing a solid experimental basis for its environmental safety assessment.

## 4 Discussion

As a broad-spectrum herbicide, glyphosate plays a pivotal role in modern cotton weed management systems due to its remarkable efficacy, environmental compatibility, and cost-effectiveness (Beckie, 2011). However, glyphosate cannot distinguish between crops and weeds, which exerts negative impacts on crop production, ultimately compromising crop quality and yield. Therefore, achieving the synergistic improvement of glyphosate resistance along with high yield and superior quality traits remains a critical objective in current crop breeding (Aoun et al., 2025). In this study, we successfully utilized multi-gene pyramiding technology to co-introduce the herbicide-resistance gene *g10evo-epsps* and the high-yield gene *csRRM2* into cotton, generating a novel transgenic line designated as L397-1 (**Figure 1**). Phenotypic analyses demonstrated that the L397–1 plants not only exhibited significantly enhanced tolerance to glyphosate but also showed increased yield and fiber length, effectively achieving the pyramiding of high resistance with high yield and superior quality. Furthermore, comprehensive environmental safety assessments of the transgenic plants, including competitive survival ability in abandoned and cultivated lands, field arthropod community structure, major field diseases, and pest population characteristics, revealed no significant differences between the transgenic L397–1 line and the recipient variety ZM24. These findings support the ecological safety and application potential of the L397–1 transgenic material for future agricultural utilization.

### 4.1 Novel breeding strategy for synergistic improvement of multiple traits

Transgenic technology has achieved tremendous success in single-trait genetic engineering breeding, with numerous gene resources conferring high yield and superior quality successfully applied to various crops, including cotton, soybean, rice, and maize (De Lange et al., 2018; Wang et al., 2022; Zhang et al., 2022; Zhang et al., 2026). However, negative correlations often exist among different traits; for instance, highly resistant cultivars frequently suffer from low yields, and early-maturing varieties often exhibit weak disease resistance. This makes the synergistic improvement of multiple elite traits a persistent challenge in crop breeding.

In recent years, significant progress has been made in pyramiding two or more favorable traits by exploiting pleiotropic factors that simultaneously regulate multiple beneficial characteristics (Iswanto et al., 2025; Li et al., 2025; Zhao et al., 2023b). Nevertheless, this strategy has so far uncovered only a few pleiotropic genes that concurrently enhance both yield and stress tolerance (Fang et al., 2019); most identified pleiotropic genes merely strengthen a single trait while inhibiting another desirable trait, thereby limiting their value in breeding (Qi et al., 2024; Wang et al., 2021). In this study, using a multi-gene co-transformation system established in our laboratory, we successfully constructed a modified plant expression vector containing a multi-gene expression cassette with both the herbicide-resistance gene *g10evo-epsps* and the high-yield gene *csRRM2* (**Figure 1**). We generated transgenic cotton lines with composite traits, thereby achieving the efficient pyramiding of high resistance, high yield, and superior fiber quality.

The tolerance of glyphosate-resistant transgenic materials to herbicides has an upper limit. However, in field conditions, high concentrations of glyphosate are often sprayed to achieve satisfactory weed control. For instance, the increased application rate may result from reduced weed susceptibility to glyphosate, elevated relative concentrations due to rapid water evaporation under high-temperature conditions, or significantly higher local concentrations caused by repeated spraying (Sammons and Gaines, 2014). Under such circumstances, glyphosate can induce phytotoxicity in cotton, potentially exerting adverse effects on yield and fiber quality. Therefore, there is an urgent need to develop cotton varieties with resistance levels exceeding the glyphosate concentrations typically applied in the field, thereby enhancing their practical value in breeding.

In this study, the transgenic line L397–1 exhibited a maximum glyphosate tolerance of up to 18× the standard field dose, with only mild phytotoxicity observed starting from the 6× concentration (**Figure 2**). Furthermore, we confirmed that the *csRRM2* gene significantly enhances both the yield and fiber length of the transgenic L397–1 cotton. Even following treatment with 6× glyphosate, the yield and quality of the transgenic line remained significantly superior to those of the control (**Figure 4**). Thus, for future application and promotion, L397–1 represents an excellent germplasm resource that simultaneously confers both herbicide resistance and high yield, making it valuable for cultivar improvement.

### 4.2 Environmental safety evaluation supports the breeding application potential of L397-1

The commercial application of transgenic crops requires rigorous environmental safety assessment to evaluate potential ecological risks. Current evaluation frameworks mainly focus on weediness potential, impacts on non-target organisms and biodiversity, and effects on pest and disease occurrence.

In this study, we systematically evaluated the competitive survival ability of L397–1 in both wasteland and cultivated habitats. The results showed no significant differences in weed coverage, cotton coverage, germination rate, and seed number per plant between L397–1 and ZM24, indicating that the transgenic line does not possess enhanced weediness or invasive potential.

Regarding biodiversity impacts, we monitored the arthropod community across 13 developmental stages in cotton fields. No significant differences were observed in biodiversity indices (Shannon-Wiener diversity, Simpson dominance, Pielou evenness) for phytophagous insects, natural enemies, and the total arthropod community. Furthermore, population dynamics of major pests (whitefly, aphid, spider mite) and the natural enemy green lacewing were comparable between L397–1 and ZM24, and pest species richness showed no significant difference. Disease surveys also revealed similar incidence levels of boll rot and Verticillium wilt between the two lines.

Taken together, these multi-dimensional assessments indicate that L397–1 has no detectable adverse ecological effects under the experimental conditions of this study, providing a safety basis for its further breeding application and environmental release.

## Data Availability

The original contributions presented in the study are included in the article/**Supplementary Material**. Further inquiries can be directed to the corresponding authors.

## Supplementary material

Supplementary Figure 1 Generation and molecular characterization of transgenic cotton L397-1. **(A)** Flowchart of the cotton genetic transformation process. **(B)** PCR positive identification of three transgenic lines. From left to right in each lane: DNA marker, CCRI 24 negative control, positive plasmid control, followed by 14 transformants. **(C)** Greenhouse transplantation and T0 generation seed harvest.

Supplementary Figure 2 Whole-genome sequencing (WGS)-based T-DNA alignment verification.

Supplementary Figure 3 Phenotypes of different transgenic materials showing glyphosate resistance. **(A)** Phenotypes of various transgenic materials observed 5 days after spraying with 41% glyphosate isopropylamine salt solution (Roundup) diluted to 2.73 g/L active ingredient under field conditions. **(B)** Phenotypes of various transgenic materials observed 5 days after spraying with 41% glyphosate isopropylamine salt solution (Roundup) diluted to 2.73 g/L a.i. under greenhouse conditions.
