# Supplementary material for: Synergistic improvement of high glyphosate resistance and high yield with environmental safety assessment
Source: Front Plant Sci. 2026 Sep 2;17:1936824. doi: 10.3389/fpls.2026.1936824 (PMC13579542; doi:10.3389/fpls.2026.1936824)
Supplement: Supplementary file 1 [file DataSheet1.pdf]

Table S1. A list of all the primers used in this study.

Primer	Sequence（5’-3’）
g10evo-epsps-538F	AGATTCCTTATGGGTGTTGC
g10evo-epsps-1285R	TGGTGTCAGAGATTCTATCGC
csRRM2-88F	GCGTGGAGGATGTCTATCTC
csRRM2-199R	AAGTTCCATTGAGACCATCG
FP1	GTAATACGACTCACTATAGGGCACGCGTGGTNTCGASTWTSGWGTT
FP2	GTAATACGACTCACTATAGGGCACGCGTGGTNGTCGASWGANAWGAA
FP3	GTAATACGACTCACTATAGGGCACGCGTGGTWGTGNAGWANCANAGA
FP4	GTAATACGACTCACTATAGGGCACGCGTGGTAGWGNAGWANCAWAGG
FP5	GTAATACGACTCACTATAGGGCACGCGTGGTNGTAWAASGTNTSCAA
FP6	GTAATACGACTCACTATAGGGCACGCGTGGTNGACGASWGANAWGAC
FP7	GTAATACGACTCACTATAGGGCACGCGTGGTNGACGASWGANAWGAA
FP8	GTAATACGACTCACTATAGGGCACGCGTGGTGTNCGASWCANAWGTT
FP9	GTAATACGACTCACTATAGGGCACGCGTGGTNCAGCTWSCTNTSCTT
FSP1	GTAATACGACTCACTATAGGGC
FSP2	ACTATAGGGCACGCGTGGT
LSP1	GCACACCACATCAGAAAGTCCTACCC
LSP2	GCTAGATTCGAATACGCTGAGGCTAC
LSP3	CCTATAGGGTTTCGCTCATGTGTTGAGC
RSP1	GTAGACAAGTGTGTCGTGCTCCACC
RSP2	GTTCGCCAGTCTTTACGGCGAGTTCTG
RSP3	CTTTGACTCCATGAAGCTAAACTGAAGG
L397-1-A11-LF	GGACCCAAACACCTTCTT
L397-T35S-LR	AGGGTAGGACTTTCTGATGTGGT
L397-T35S-F1	ACCACATCAGAAAGTCCTACCCT
L397-T35S-R1	GGAATTAGAAATTTTATTG
g10evo-epsps-F	AGTTAGCAGAATCTGCAATGGT
g10evo-epsps-R	CGAGCACTCTACCTGGTCTG
L397-g10P35S-F1	CAAGTGGATTGATGTGATAACATG
L397-g10P35S-R1	ACGATGAAGAAATTGGATAAGCT
L397-Tnos-F1	GTTAAATATTCAAGCAAAGAGACG
L397-Tnos-R1	AGTAACATAGATGACACCGCGC
csRRM2-F	CAAGCCACTGAAAACGAGGT
csRRM2-R	GGCAACAGGATTCAATCTTAAG
L397-P35S-F1	TTATTGTGAAGATAGTGGAAAAGG
L397-P35S-R1	AACCTCGTTTTCAGTGGCTTG
L397-1-A11-RF	GCGAGTTCTGTTAGGTCC
L397-1-A11-RR	TATTGTGCGTGAAGTTGT
qRT-PCR-g10F	ACGAGCTTATCCAATTTCTT
qRT-PCR-g10R	GTTCTTGGATGGCTGTGCTC
qRT-PCR-RRM2F	CAAGCCACTGAAAACGAGGT

qRT-PCR-RRM2R	CATGGTATAAGTTCCATTGA
qRT-PCR-ActinF	CAAGGAAATTACTGCCTTGG
qRT-PCR-ActinR	CTAAGATGGAGCCTCCAATC
Southern-epsps-F	GACACCCAAGCACCAAGT
Southern-epsps-R	GCACAGCCATCCAAGAAC
