# Supplementary material for: Synergistic improvement of high glyphosate resistance and high yield with environmental safety assessment
Source: Front Plant Sci. 2026 Sep 2;17:1936824. doi: 10.3389/fpls.2026.1936824 (PMC13579542; doi:10.3389/fpls.2026.1936824)
Supplement: Supplementary file 2 [file SupplementaryFile1.docx]

**Supplementary Figure**

**Fig. S1. Generation and molecular characterization of transgenic cotton L397-1.**
**(A)** Flowchart of the cotton genetic transformation process. **(B)** PCR positive identification of three transgenic lines. From left to right in each lane: DNA marker, CCRI 24 negative control, positive plasmid control, followed by 14 transformants. **(C)** Greenhouse transplantation and T0 generation seed harvest.

**Fig. S2 Whole-genome sequencing (WGS)-based T-DNA alignment verification**

**Fig. S3 Phenotypes of different transgenic materials showing glyphosate resistance**

**(A)** Phenotypes of various transgenic materials observed 5 days after spraying with 41% glyphosate isopropylamine salt solution (Roundup) diluted to 2.73 g/L active ingredient under field conditions. **(B)** Phenotypes of various transgenic materials observed 5 days after spraying with 41% glyphosate isopropylamine salt solution (Roundup) diluted to 2.73 g/L a.i. under greenhouse conditions.
